# Expression of Testis Specific Genes TSGA10, TEX101 and ODF3 in Breast Cancer

**DOI:** 10.5812/ircmj.3611

**Published:** 2012-11-15

**Authors:** Mehdi Dianatpour, Parvin Mehdipour, Karim Nayernia, Maryam-Beigom Mobasheri, Soudeh Ghafouri-Fard, Shahram Savad, Mohammad Hossein Modarressi

**Affiliations:** 1 Department of Medical Genetics, School of Medicine, Tehran University of Medical Sciences, Tehran, IR Iran; 2 Institute of Human Genetics, North East England, Stem Cell Institute, International Center for Life, Newcastle University, Newcastle, UK; 3 Department of Medical Genetics, School of Medicine, Shahid Beheshti University of Medical Sciences, Tehran, IR Iran

**Keywords:** Testis, Gene, TSGA10, TEX101, ODF3, Breast Cancer

## Abstract

**Background:**

Breast cancer is the most common malignancy in women around the world so finding new biomarkers for early detection and also study on molecular aspects of breast cancer is valuable. Cancer testis genes are a group of genes expressed solely in testis and in a range of human malignancies.

**Objectives:**

In this study we determined the expression of cancer testis genes Tsga10, TEX101 and ODF3 in patients with breast cancer.

**Materials and Methods:**

Fifty patients with breast cancer were enrolled in this study. Breast cancer cell lines MCF-7 and MDA-MB-231 were also used to determine the expression of testis cancer genes. For both patients and cell lines, cancer testis genes of TSGA10, TEX101 and ODF3 were determined by RT-PCR. The presence of auto antibody against these genes in patients’ serums was carried on by ELISA method.

**Results:**

Seventy percent of patients showed TSGA10 expression but none of them showed expression of TEX101 and ODF3. Fourteen percent of patients were positive for anti TSGA10 but all patients were negative for anti TEX101 and anti ODF3. Both of breast cancer cell lines exhibited very strong expression of TSGA10.

**Conclusions:**

Because of the important roles of Tsga10 in cell proliferation, we concluded that this gene may have a role in proliferation and survival of breast cancer cells and could be used for diagnosis and immunotherapy of breast cancer.

## 1. Background

Breast cancer is the most prevalent malignancy in women and affects about 1 in 8 women around the world (1). Therefore investigation on early detecting biomarkers and also study on molecular aspects of breast cancer for improvement of breast cancer therapy is valuable.

Cancer testis genes are a group of genes predominantly expressed in male germinal cells (2, 3). They have no expression or very slight expression in other normal somatic tissues but maybe aberrantly expressed in various human cancers (4-7). So far more than 100 cancer testis genes have been identified, some of them located on X chromosome and referred to CT-X genes and the others located on other chromosomes (8). CT-X antigen expression is associated with a poorer outcome and is more prevalent in higher grade and advanced stage tumors (9). Due to testis blood barrier and the immune privileged status of germinal cells, (10) expression of CT genes in tissues other than testis can trigger immune response. They can be considered as tumor specific markers and represent ideal targets for cancer vaccines and cancer immunotherapy. In addition, some clinical trials currently were carried out in this regards (11).

## 2. Objectives

In this study we tried to show the expression of three cancer testis genes, TSGA10, TEX101 and ODF3 in breast cancer patients as well as breast cancer cell lines. We also investigate the presence of auto antibodies against them in patients’ sera.

## 3. Materials and Methods

### 3.1. Tissue and Serum Samples

Breast cancer tissues and serum samples were obtained from tumor bank of cancer institute Imam Khomeini hospital under the protocols of Medical Ethics Committee. All patients had written informed consent. Fifty tumor tissues and 50 adjacent noncancerous tissue (ANCT) samples as normal breast tissue were obtained (Table 1). Ten fibroadenoma samples also obtained for comparison between malignant and benign tumor tissues. Normal testis tissues were obtained from a prostate cancer patient following orchiectomy and used as positive control for testis specific genes expression. Normal serums were collected from 50 normal healthy women.

**Table 1:** Pathological and HER2 Characteristics of Patients

	Tumor	ANCT a
**Sample**	50	50
**Age**	37-68 Mean:53	
**Histology**		
**Ductal**	46	
**Others**	4	
**Grade**		
**1**	10	
**2-3**	40	
**HER2/neu**		
**Negative**	37	
**Positive**	13	

^a^Abbreviations: ANCT: Adjacent non-cancerous tissue

### 3.2. Cell Culture

The human breast cancer cell lines MDA-MB231 and MCF-7 were obtained from Pasteur Institute of Iran and cultured according to the manufacturer’s instruction. Briefly, cells cultured in RPMI medium 10% FBS at 37°C and 5% co2. After two days, cells harvested, counted and 2x10⁶ cells were separated for RNA extraction, cDNA synthesis and RT-PCR.

### 3.3. Total RNA Extraction and cDNA Preparation

Total RNA was extracted from frozen tumor samples and breast cancer cell lines using Tripure [Rosch] according to the manufacturer’s instructions. RNA was dissolved in DEPS-treated water and concentration was determined by spectrophotometer (Nano drop 2000). About 1-5 µg of total RNA of various samples were used to carry out cDNA synthesis with reverse transcription kit (Fermentase).

### 3.4. RT-PCR and Semi Nested PCR

Amplification reaction carried out using following primers and conditions. Amplification of the housekeeping gene, GAPDH was used to check the quality of cDNA. All primers designed so that forward and reverse primers attached to different exons of each gene to avoid false positive because of probable DNA contamination during RNA extraction. In order to determine the exact expression of each gene and determine low level expression of genes, amplification of cDNA was done in two steps. The first PCR carried out using F1 and R1 primers and semi nested PCR by F2 or R2 primers using 1 µL of the first pcr product. Finally, PCR products were separated on 2% agarose gel and then visualized under UV light after DNA staining.

ODF3 F: 5'-CAGTGAGCTCCATGACG-3'

ODF3 R1: 5'-GCAGGGCTGGCGTTATTCC-3'

ODF3 R2: 5'-GTAGTCACCTGGACCAGGAG-3'

2 min at 95 ºC, followed by 30 cycles of 30 s at 95 ºC, 30 s at 57 ºC, and 80 s at 72 ºC, 5 min at 72 ºC

TEX101 F1: 5'-GGCAGATCCAGACCAGCTCC-3'

TEX101 R: 5'- TGCCACCTCCAGTGATCTCAAG-3'

TEX101 F2: 5'-

GGGAGTTCAGTGAGACCACAG-3'

2 min at 95 ºC, followed by 30 cycles of 30 s at 95 ºC, 30 s at 60ºC, and 80 s at 72 ºC, 5 min at 72 ºC

TSGA10 F1: 5'- CAAGACGCCCATCACCAACTG-3'

TSGA10 F2: 5'- CAACGGCACATGCTATTCTCC-3'

TSGA10 R: 5'- CCACAGTGCTTATGGTTTCCTTC-3'

2 min at 95 ºC, followed by 30 cycles of 30 s at 95 ºC, 30 s at 60ºC, and 50 s at 72 ºC, 5 min at 72 ºC

### 3.5. Recombinant Protein Production and ELISA

To determine antibody against TAGA10, TEX101 and ODF3 in sera of the patients and normal healthy control, ELISA test was carried out. Briefly, Total length of TEX101 and ODF3 cDNA and 400 bp cDNA from N terminal of TSGA10 were cloned in expression vector pmal c2x. The recombinant proteins were expressed and purified. The purity of the protein analyzed by SDS-PAGE. ELISA plates were coated by 50µl /well of 1µg/ml purified TEX101 protein in coating buffer (carbonate buffer, PH 9.6) and incubated at 4°C for overnight and then washed 2x with PBS-0.05% tween 20(PBS-T). After coating, plates were blocked by 200 µl /well of 5% non-fat dry milk in PBS-T for 1 hour at 37°C. Fifty µl of different dilutions of patients and controls serums in 1% non-fat dry milk in PBS-T were added and incubated at room temperature for 1 h and washed 3X by PBS-T. The best results were obtained with sera dilution of 1/600. Horseraddish peroxidase- conjugated goat anti human Ab added as secondary Ab (1/15000 in 1% non-fat dry milk in PBS-T, 50 µl /well) and incubated 1h at room temperature (RT). Plates were shacked off and washed 3x with PBS-T and 50 µl of TMB substrate (Padtan danesh) were added in each wells and incubated 15 min at RT in dark room. Color production stopped by stopping solution (5N H2SO4, 50 µl /well) and the absorbance was determined at 450 nm with 620nm as reference wavelength. Sera of fifty normal healthy donors were tested as control, and results above the controls mean absorbance ± 2SD, considered positive. All patients and controls sera were tested in duplicates.

## 4. Results

**Figure 1:**
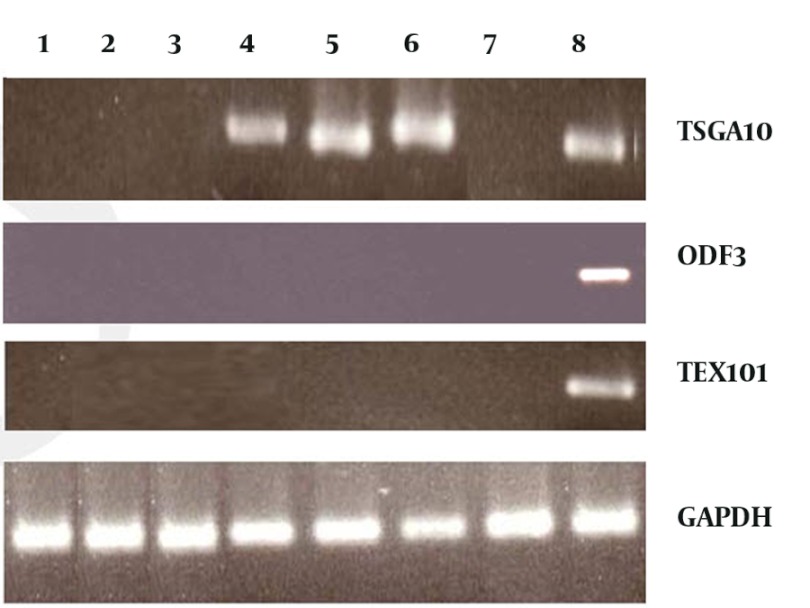
First round PCR of testis specific genes Lane 1-4: Breast cancer patients, 5: MCF-7, 6: MDA-231, 7: Fibroadenoma 8: Positive control (testis)

**Figure 2:**
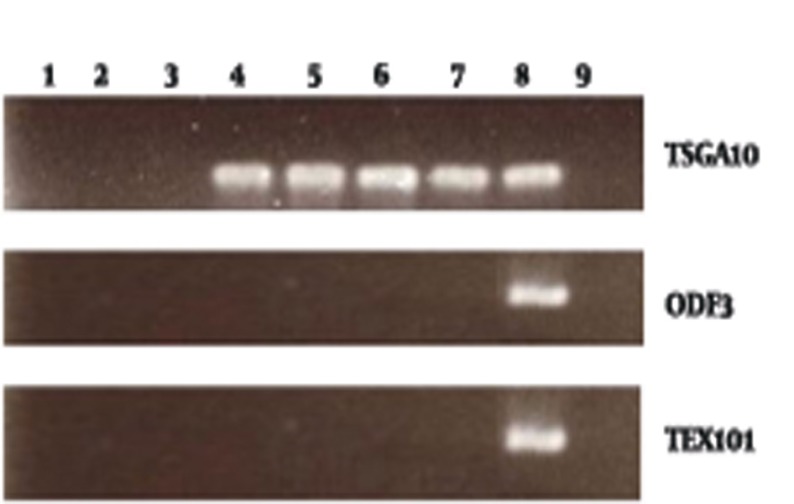
Seminested PCR of testis specific genes 1,2 ANCT , 3 Fibroadenoma, 4-7 Breast cancer patients , 8 Positive control, 9 H2O

RT-PCR of patients’ tumor samples and breast cancer cell lines were carried out using specific primers for TSGA10, PIWIL2, TEX101 and ODF3 genes. Different level of gene expression was present in tumor samples and breast cancer cell lines (Figure 1). In order to analyze the level of gene expression, semi quantitative expression analysis was carried out by semi-nested RT-PCR (Figure 2).

### 4.1. TSGA10

TSGA10 is expressed predominantly in testis and in some tumors.In our study TSGA10 expressed in 35/50 (70%) of breast cancer samples, in which 5 (10%) showed expression in the first RT-PCR, and 30 (60%) in the reamplification, semi nested PCR. No expression of TSGA10 was shown in ANCT and fibroadenoma samples. There was no correlation between type and grade of tumor and expression of TSGA10. We analyzed the expression of TSGA10 in breast cancer cell lines. Both breast cancer cell lines (MDA-231 and, MCF-7) showed high expression of the gene in the first round of RT-PCR.

### 4.2. TEX101 and ODF3

None of breast cancer samples and cell lines showed expression of TEX101 and ODF3. Semi nested PCR was carried out on RT-PCR products but all of them was negative. ANCT and fibroadenoma tissues also were negative for TEX101 and ODF3 expression.

### 4.3. ELISA

ELISA test was performed for detection of antibody against TSGA10, TEX101 and ODF3 in serum of breast cancer patients and normal healthy controls. ELISA test for TSGA10 was positive in 6/50 (12%) of breast cancer patients, but it was negative in all normal control serums. ELISA test for TEX101 and ODF3 was negative in all breast cancer and normal control serums (Figurs 3,4,5).

**Figure 3:**
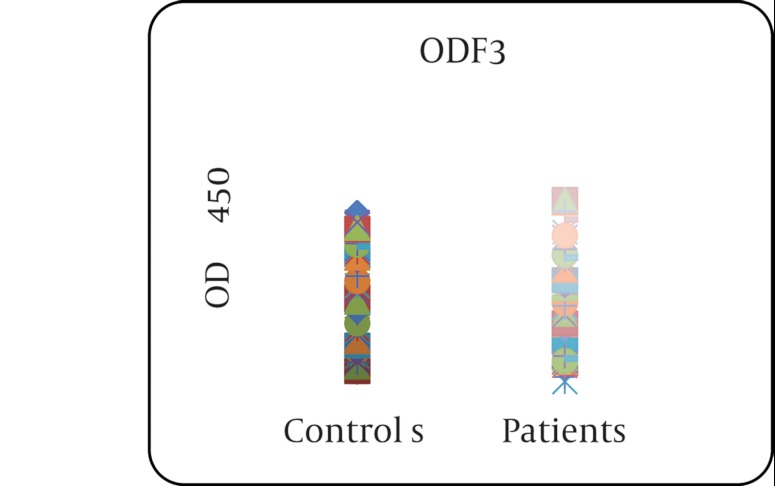
ELISA test for anti-ODF3 antibody in breast cancer patients

**Figure 4:**
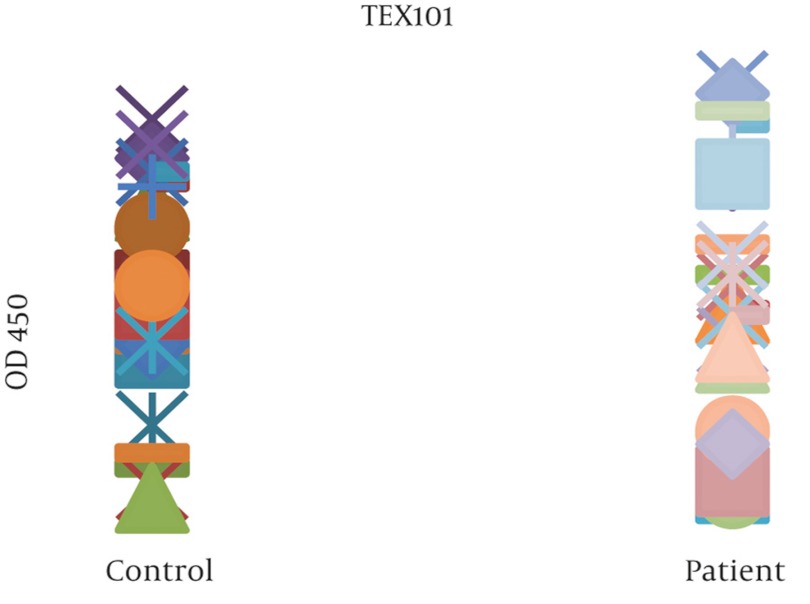
ELISA test for anti-TEX101 antibody in breast cancer patients

**Figure 5:**
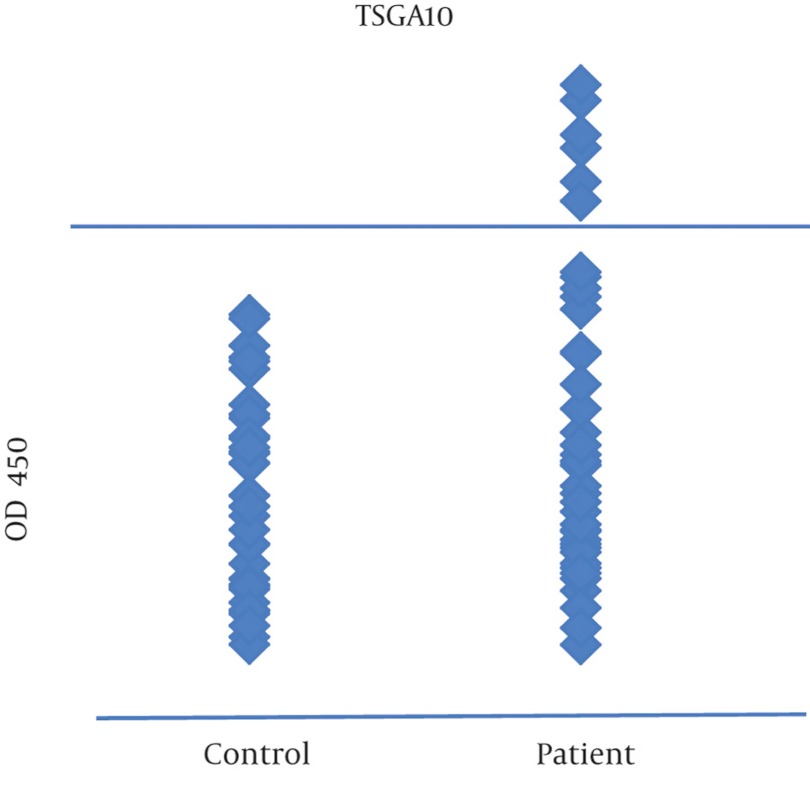
ELISA test for anti-TSGA10 antibody in breast cancer patients

## 5. Discussion

Breast cancer is the most prevalent cancer between women and early detection of cancer is so important for better management and treatment of the patients. Development of new molecular tests and markers for early detection of cancers and also new strategy with high efficiency for cancer treatment are very important requirements. Some tumor markers such as CA 15-3 were introduced as a serum breast cancer marker but they are expressed in late stage of cancer and only a minority of patients expresses them at early stages of breast cancer.

Cancer testis antigenes are a group of tumor antigens that expressed specifically in testis and also in various cancers but not in normal somatic tissues (12). There are several studies that showed the expression of some CT antigens such as NY-ESO, MAGEA and CT-10 in breast cancer (13).

Testis has a special feature called blood–testis barrier and it is formed by tight connections between Sertoli cells. Blood test is barrier provides an immune privileged area for germ cells and immune system has no access to germ cells and if for any reason testis antigens entered the blood stream, they can produce an autoimmune response. This special feature made CT antigens a promising tumor specific marker and a very good candidate for cancer vaccines and immunotherapy.

There are several clinical trials of immunization against CT antigens such as MAGE and CTAG1 in various cancers. Recently we have several studies on CT antigens and we identified a novel gene, TSGA10 (14, 15). Human TSGA10 expressed in normal testis. In addition, TSGA10 expression has been demonstrated in embryonic stem cell, in actively dividing cells, in fetal differentiating tissues and also in various primary tumors, (16) therefore it could be classified as CT antigen. Previously, our studies showed expression of TEX101, SPATA19 and LEMD1 in basal cell carcinoma (17) and prostate cancer (18, 19).

In conclusion, the expression of three CT antigens, TSGA10, TEX101 and ODF3 were checked in breast cancer patients. Expression of TSGA10 in 70% of patients with breast cancer and the presence of auto Ab against TSGA10 in 12% of patients confirmed the immune response against CT antigens in the patients. Therefore, it may suggest the possibility of application of this gene for breast cancer vaccines, immunotherapy and also can be considered as a tumor marker.

## References

[1] Jemal A, Siegel R, Ward E, Hao Y, Xu J, Thun MJ (2009). Cancer statistics, 2009.. CA Cancer J Clin.

[2] Almeida LG, Sakabe NJ, deOliveira AR, Silva MC, Mundstein AS, Cohen T (2009). CTdatabase: a knowledge-base of high-throughput and curated data on cancer-testis antigens.. Nucleic Acids Res.

[3] Hofmann O, Caballero OL, Stevenson BJ, Chen YT, Cohen T, Chua R (2008). Genome-wide analysis of cancer/testis gene expression.. Proc Natl Acad Sci U S A.

[4] Karn T, Pusztai L, Ruckhaberle E, Liedtke C, Muller V, Schmidt M (2012). Melanoma antigen family A identified by the bimodality index defines a subset of triple negative breast cancers as candidates for immune response augmentation.. Eur J Cancer.

[5] Adams S, Greeder L, Reich E, Shao Y, Fosina D, Hanson N (2011). Expression of cancer testis antigens in human BRCA-associated breast cancers: potential targets for immunoprevention?. Cancer Immunol Immunother.

[6] Chen YT, Ross DS, Chiu R, Zhou XK, Chen YY, Lee P (2011). Multiple cancer/testis antigens are preferentially expressed in hormone-receptor negative and high-grade breast cancers.. PLoS One.

[7] Ghafouri-Fard S, Modarressi MH (2009). Cancer-testis antigens: potential targets for cancer immunotherapy.. Arch Iran Med.

[8] Simpson AJ, Caballero OL, Jungbluth A, Chen YT, Old LJ (2005). Cancer/testis antigens, gametogenesis and cancer.. Nat Rev Cancer.

[9] Zhou JX, Li Y, Chen SX, Deng AM (2011). Expression and prognostic significance of cancer-testis antigens (CTA) in intrahepatic cholagiocarcinoma.. J Exp Clin Cancer Res.

[10] Pelletier RM, Byers SW (1992). The blood-testis barrier and Sertoli cell junctions: structural considerations.. Microsc Res Tech.

[11] (2012). Clinical Trials.gov A service of the U.S. National Institutes of Health.. http://www.clinicaltrials.gov.

[12] Scanlan MJ, Simpson AJ, Old LJ (2004). The cancer/testis genes: review, standardization, and commentary.. Cancer Immun.

[13] Curigliano G, Viale G, Ghioni M, Jungbluth AA, Bagnardi V, Spagnoli GC (2011). Cancer-testis antigen expression in triple-negative breast cancer.. Ann Oncol.

[14] Lee JH, Jung C, Javadian-Elyaderani P, Schweyer S, Schutte D, Shoukier M (2010). Pathways of proliferation and antiapoptosis driven in breast cancer stem cells by stem cell protein piwil2.. Cancer Res.

[15] Modarressi MH, Cameron J, Taylor KE, Wolfe J (2001). Identification and characterisation of a novel gene, TSGA10, expressed in testis.. Gene.

[16] Mobasheri MB, Jahanzad I, Mohagheghi MA, Aarabi M, Farzan S, Modarressi MH (2007). Expression of two testis-specific genes, TSGA10 and SYCP3, in different cancers regarding to their pathological features.. Cancer Detect Prev.

[17] Ghafouri-Fard S, Abbasi A, Moslehi H, Faramarzi N, Taba Taba Vakili S, Mobasheri MB (2010). Elevated expression levels of testis-specific genes TEX101 and SPATA19 in basal cell carcinoma and their correlation with clinical and pathological features.. Br J Dermatol.

[18] Ghafouri-Fard S, Ousati Ashtiani Z, Sabah Golian B, Hasheminasab SM, Modarressi MH (2010). Expression of two testis-specific genes, SPATA19 and LEMD1, in prostate cancer.. Arch Med Res.

[19] Hagtvedt T, Aalokken TM, Notthellen J, Kolbenstvedt A, Haye R (2003). [Sinus radiography and low-dose CT in the diagnosis of acute sinusitis. ].. Tidsskr Nor Laegeforen.

